# A Label-Free Electrochemical Immunosensor for Carbofuran Detection Based on a Sol-Gel Entrapped Antibody

**DOI:** 10.3390/s111009520

**Published:** 2011-10-10

**Authors:** Xia Sun, Shuyuan Du, Xiangyou Wang, Wenping Zhao, Qingqing Li

**Affiliations:** School of Agriculture and Food Engineering, Shandong University of Technology, NO.12, Zhangzhou Road, Zibo 255049, Shandong Province, China; E-Mails: sunxia2151@sina.com (X.S.); dushuting19871229@126.com (S.D.); 187286528zwp@163.com (W.Z.); liqing929929@163.com (Q.L.)

**Keywords:** immunosensor, amperometric, label-free, sol-gel, carbofuran

## Abstract

In this study, an anti-carbofuran monoclonal antibody (Ab) was immobilized on the surface of a glassy carbon electrode (GCE) using silica sol-gel (SiSG) technology. Thus, a sensitive, label-free electrochemical immunosensor for the direct determination of carbofuran was developed. The electrochemical performance of immunoreaction of antigen with the anti-carbofuran monoclonal antibody was investigated by cyclic voltammetry (CV) and electrochemical impedance spectroscopy (EIS), in which phosphate buffer solution containing [Fe(CN)_6_]^3−/4−^ was used as the base solution for test. Because the complex formed by the immunoreaction hindered the diffusion of [Fe(CN)_6_]^3−/4−^ on the electrode surface, the redox peak current of the immunosensor in the CV obviously decreased with the increase of the carbofuran concentration. The pH of working solution, the concentration of Ab and the incubation time of carbofuran were studied to ensure the sensitivity and conductivity of the immunosensor. Under the optimal conditions, the linear range of the proposed immunosensor for the determination of carbofuran was from 1 ng/mL to 100 μg/mL and from 50 μg/mL to 200 μg/mL with a detection limit of 0.33 ng/mL (S/N = 3). The proposed immunosensor exhibited good high sensitivity and stability, and it was thus suitable for trace detection of carbofuran pesticide residues.

## 1. Introduction

Pesticides are widely used in agriculture to protect seeds and crops, which leads to them being among the most important environmental pollutants and the cause of severe impairment of human health. At present, classical analytical techniques [*i.e.*, gas chromatography (GC), high-pressure liquid chromatography (HPLC), capillary electrophoresis (CE) and mass spectrometry (MS)] are very sensitive and standardized techniques for the analysis of pesticide residues [1–3]. However, they have some disadvantages such as complexity, extensive time consumption, the need for costly, bulky instrumentation and so on [4,5]. For these reasons, the development of rapid and efficient monitoring methods for recognitive and quantitative detection of the presence of pesticide residues in food becomes more and more important.

Enzyme-linked immunosorbent assays (ELISAs) have gained a place on the analytical benchtop as alternative or complementary methods for routine pesticide analysis. They are fast, economic, and at least as sensitive as usual chromatographic techniques. However, analyte detection in ELISAs needs to label one of the immunoreagents. Moreover, ELISAs need extensive sample handling such as rather large number of washing steps [6]. On the contrary, for immunosensors, one of the immunoreagents is immobilized on the surface of the transducer, and a direct physical signal is produced when the immunochemical interaction occurs. This label-free detection represents an essential advantage of immunosensors as compared to label-dependent immunoassays [7].

Immunosensors are biosensors that use antibodies (Ab) or antigens (Ag) as the specific sensing element and provide concentration-dependent signals [8]. Depending on the method of signal transduction, immunosensors may be divided into four basic groups: electrochemical, optical, piezoelectric and thermometric [9]. Among these different types of immunosensors, the electro-chemical ones show more potential thanks to their higher sensitivity, higher speed and permanent control. Valera’s group [10–15] has reported a series of methods using electrochemical impedance immunosensors for atrazine detection based on the interdigitated microelectrode array produced by a competitive reaction. The Ciumasu group also reported a portable miniaturized immunosensor for atrazine and diuron detection using a competition assay method [16]. It is well known that competitive assays are complex. The direct determination of a pesticide by immobilizing a suitable antibody on the electrode surface could simplify the assay process and shorten the detection time. Hu *et al.* reported a label-free electrochemical immunosensor based on gold nanoparticles for the direct determination of paraoxon. The recovery of paraoxon in river water using the developed immunosensor ranged between 93.5–109% [6]. For electrochemical immnunosensors, because pesticides are small molecule compounds, in fact, the electro-signal change due to the immunoreaction is usually faint. Thus, the use of direct immunoreactions for pesticide detection is still a challenge.

During the fabrication of an immunosensor, the immobilization of the antigen or antibody onto the electrode surface is a difficult and crucial step, which heavily influences the performance of the resulting immunosensor. At present the main immobilization approaches include the electropolymerization entrapment technique using, for example, a polypyrrole monolayer film [17], polypyrrole/polybilayer film [18], self-assembled monolayers [19], or sol-gel film [20] *etc*. Sol-gel technology provides a unique means to prepare a three-dimensional network suited for the encapsulation of a variety of biomolecules. Thus, here, we select silica gel sol gel (SiSG) as immobilizing agent to offer a biocompatible microenvironment for confining the antibody and foreshadow the great potentiality of this immobilizing agent in the development of biosensors [21]. To the best of our knowledge, such a label-free, direct immunoreaction immunosensor with a silica SiSG immobilized antibody for carbofuran pesticide detection has not yet been reported.

In this work, a label-free electrochemical immunosensor was developed as a tool for carbofuran detection. Because SiSG possesses a silicate network and provides a biocompatible microenvironment around the antibody, the antibody is efficiently absorbed onto the surface of glassy carbon electrode (GCE). The performance of the immunoreaction was characterizated by cyclic voltammetry (CV) and electrochemical impedance spectroscopy (EIS). The main parameters affecting the electrochemical responses were optimized, including operating pH, the concentration of Ab and incubation time. The proposed immunosensor exhibited good accuracy, high sensitivity and a wide linear range with a low detection limit.

## 2. Materials and Methods

### 2.1. Apparatus

Cyclic voltammetry was performed with a CHI660D electrochemical workstation (Shanghai Chenhua Co., China). The electrochemical impedance spectroscopy (EIS) measurements were carried out using a Model IM6e unit (ZAHNER Elektrick Co., Germany).The working electrode was a glassy carbon electrode (GCE, d = 3 mm), an Ag/AgCl (saturated KCl) and platinum electrode were used as reference and auxiliary electrodes, respectively. If not mentioned, all potentials given below were relative to Ag/AgCl (saturated KCl) electrode.

### 2.2. Reagents

Anti-carbofuran monoclonal antibody, carbofuran, and Tween 20 were all purchased from Sigma. Tetraethoxysilane (TEOS) was obtained from Tianjin Hengxing Co. (China). 0.01 M Phosphate buffer solution (PBS, pH 7.4, high-pressure sterilization) was used for dissolving the anti-carbofuran monoclonal antibody. 0.01 M PBS (pH 7.0) containing 5 mmol/L of K_3_[Fe(CN)_6_]/K_4_[Fe(CN)_6_] (1:1 mixture as redox probe) and 0.1 M KCl was used as substrate solution. All other reagents were of analytical grade.

### 2.3. Preparation of the Immunosensor

The whole experimental procedure can be summarized in four steps: electrode cleaning, SiSG preparation, antibody immobilization and pesticide detection. Step 1: GCE was sonicated in a hot mixture of “piranha solution” (a mixture of concentrated H_2_SO_4_ and 30% H_2_O_2_ at the volume ratio of 3:1), rinsed with deionized water, and dried in air. The GCE was carefully polished using alumina slurries with particle diameter 0.5 μm and 50 nm, respectively, and rinsed with deionized water. Then it was immersed in 6 M HNO_3_, absolute ethanol and deionized water in an ultrasonic bath for 5 min, respectively, followed by electrochemical etching in 0.5 mol/L H_2_SO_4_ using a cycling electrode potential from −1.0 to 1.0 V (versus SCE) at a scan rate of 100 mV/s until stabilization. The electrode was further cleaned and activated, and then dried for use. Step 2:2 mL of TEOS, 1 mL of ultrapure water, 25 μL of HCl (0.1 mol/L) and 50 μL of Tween 20 were mixed in an ultrasonic bath for an hour until the sol was clear and transparent, then the sol was refrigerated for another 2 hours [22]. Step 3: After 0.5 mL of SiSG and 0.5 mL of anti-carbofuran monoclonal antibody were mixed under magnetic stirring for 10 min, the immobilization of Ab was completed by dropping a 5 μL of the above mixture onto the pretreated GCE surface, and it was then stored at 4 °C for use. Step 4: For the immunoreaction, the Ab/SiSG/GCE was incubated in 20 mL of PBS (pH 7.5) containing different concentrations of carbofuran at 37 °C for 20 min.

### 2.4. Electrochemical Measurements

Cyclic voltammetry (CV) and electrochemical impedance spectroscopy (EIS) were performed in 10 mL of 0.01M PBS (pH 7.0) containing 5 mmol/L of K_3_[Fe(CN)_6_]/K_4_[Fe(CN)_6_] (1:1 mixture as redox probe) and 0.1 M KCl at room temperature. The cyclic voltammetry was performed in the working phosphate buffer (pH 7.0) at scan rate of 100 mV/s. The impedance spectra was measured in the frequency range from 0.05 to 10^4^ Hz in a potential of 0.15 V *versus* Ag/AgCl (saturated KCl), with a voltage amplitude of 5 mV. The carbofuran detection was based on the variation of current response (Δ*I* = *I_0_* − *I_1_*) before and after immunoreaction, where *I_0_* and *I_1_* were the sensors responses before and after immunoreaction to the carbofuran, respectively.

### 2.5. Preparation and Determination of Real Samples

Cabbage and lettuce were purchased from a supermarket and cleaned three times using double-distilled water. Different concentrations of carbofuran solution were sprinkled on the surface of cabbage and lettuce [23]. After 24 h, samples weighing 10 g were chopped and meshed. Then a mixed solution of 1 mL acetone and 9 mL 0.1 M phosphate buffer (pH 7.5) were added to each sample. All the above experiments were maintained under a nitrogen atmosphere and the mixed solution was treated under ultrasound for 20 min. The suspensions were centrifuged (10 min, 10,000 rpm) and the supernatants were directly detected by CV without any extraction or preconcentration.

## 3. Results and Discussion

### 3.1. Cyclic Voltammetry Characterization

**Figure 1.**
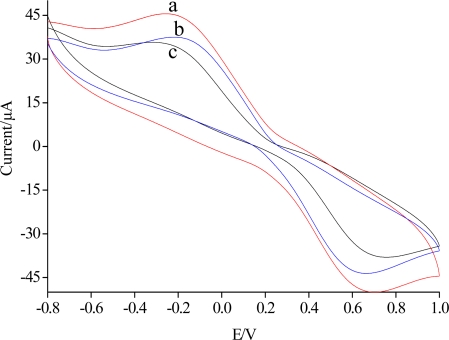
Cyclic voltammograms of SiSG/GCE (a) and Ab/SiSG/GCE (b), Ab/SiSG/GCE incubated in 10 μg/mL of carbofuran for 20 min (c) in the presence of 0.01 M pH 7.0 PBS and 5.0 mmol/L [Fe(CN)_6_]^3−/4−^. The scan rate was 100 mV/s.

Figure 1 shows the cyclic voltammograms of SiSG/GCE,Ab/SiSG/GCE and carbofuran/Ab/SiSG/GCE in the presence of 0.01 M PBS (pH 7) and 5.0 mmol/L [Fe(CN)_6_]^3−/4−^ at a scan rate of 100 mV/s, respectively. The cyclic voltammograms of SiSG/GCE (curve a) exhibited a defined reversible redox behavior attributed to high electron-transfer between [Fe(CN)_6_]^3−/4−^ solution and the electrode which was modified with SiSG with a three-dimensional network mass. With the attachment of the Ab/SiSG film on the surface of the electrode, the magnitude of current decreased (curve b) due to the complex of silica gel and antibody partially blocking the electron transfer between [Fe(CN)_6_]^3−/4−^ solution and the electrode. Similarly, after Ab/SiSG/GCE was incubated in 0.01 M PBS (pH 7.5) containing a certain concentration of carbofuran pesticide, the magnitude of current was further decreased (curve c), which also indicated the electron transfer blocking action of Ab/SiSG. The formed immuno-complex on the surface of the Ab/SiSG/GCE further blocked electron-transfer between [Fe(CN)_6_]^3−/4−^ solution and the electrode, which led to a decrease of the current of the carbofuran/Ab/SiSG/GCE sensor. This clearly suggested that the SiSG plays a crucial role as immobilizing agent for Ab, allowing them to retain their native structure and consequently also their bioelectrochemical properties, and indicated a very good permeability of the SiSG layer to [Fe(CN)_6_]^3−/4−^ [24].

### 3.2. Impedance Spectroscopy Characterization

**Figure 2.**
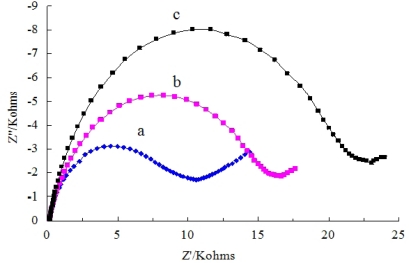
Impendance spectra corresponding to SiSG/GCE in 5 mmol/L [Fe(CN)_6_]^3−/4−^ solution: (a), the Ab/SiSG/GCE before (b) and after (c) incubation in 10 μg/mL of carbofuran in the presence of 0.01 M PBS (pH 7.0) solution and 5 mmol/L [Fe(CN)_6_]^3−/4−^.

The detailed electron-transfer behaviors of Ab/SiSG/GCE were also examined by electrochemical impedance spectroscopy. The clear semicircle portions were recorded in Nyquist plots of electrochemical impedance spectra (Figure 2) for the SiSG/GCE (curve a), Ab/SiSG/GCE (curve b), and carbofuran/Ab/SiSG/GCE (curve c), respectively.

The electron-transfer resistances of the redox (Ret) for Ab/SiSG/GCE obviously increased after incubation with carbofuran (curve c). The increase of Ret was caused by electrically insulating bioconjugates produced from specific interaction of carbofuran and Ab, which will block the electron-transfer of [Fe(CN)_6_]^3−/4−^ solution to the electrode. The results were in agreement with the conclusions obtained from the CV data.

### 3.3. Optimization of Operating Conditions

**Figure 3.**
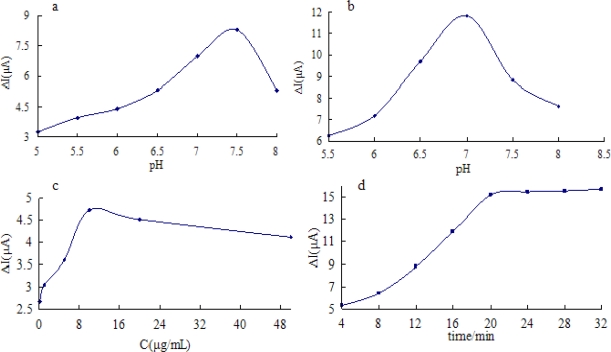
Optimization of experimental parameters: the effect of the pH of the incubation solution on the current response of Ab/SiSG/GCE (a); the effect of the pH of working solution on the current response of Ab/SiSG/GCE (b); the effect of the concentration of Ab on the current response of Ab/SiSG/GCE (c); the effect of the incubation time on the current response of Ab/SiSG/GCE (d).

The pH of the incubation solution can affect the immunoreaction between Ab and carbofuran. As shown in Figure 3a, the effect of pH was studied using CV in a series of incubating solutions with pH values ranging from 5.5 to 8.0. The minimum peak current can be obtained at pH 7.5. The reason may be that the Ab was not very stable in acid or alkaline solutions. Therefore, it was suitable to immunoreaction. Thus, the pH of incubation solution was selected as 7.5 in the subsequent experiments.

The pH of the working phosphate buffer is an important influencing factor for the sensitivity of immunosensors. Figure 3b illustrates ΔI of carbofuran/Ab/SiSG/GCE in working solutions of different pH in the range from 5.0 to 8.0. The current responses increased with the increase of pH from 5.5 to 7.0, and then decreased as pH increased further. The reason for that was the antigen-antibody complex could easily be dissociated in a working solution with unsuitable pH. Obviously, pH value had a significant influence on the current value of the Ab/SiSG/GCE sensor. The pH 7.0 of PBS was used as working solution in the subsequent experiment. The effect of the concentration of Ab on the current response was also investigated. Figure 3c shows the effect of the concentration of Ab captured in SiSG on the current response of Ab/SiSG/GCE. The current change (ΔI) curve of Ab/SiSG attaching on the surface of GCE before and after was recorded with a different Ab concentration. The ΔI increased with the increasing of the Ab concentration ranges from 0 to 10 μg/mL, and then rapidly decreased as the Ab concentration increased further. This was possibly due to overloading of the support layers which can lead to the aggregation of the antibody molecules [25]. In the other hand, the biospecific antibody binded onto the sensor surface existed competition, which led to a reduction of the number of available antibody binding sites for capturing the carbofuran. Therefore, an antibody loading of 10 μg/mL was chosen in all following experiments.

The influence of incubation time on response signals was also investigated. The immunosensor was incubated with a standard carbofuran solution (10 μg/mL) for different times from 4 to 32 min, and then tested in 0.01 M PBS (pH 7.0). Because the immunoreaction of the antigens and Ab needs some time to form immunocomplexes [6], as shown in Figure 3d, the ΔI increased with increasing incubation time and reached a maximum value at 20 min and after that the variation slowed, indicating a saturated binding of the immobilized Ab and carbofuran. Thus, the incubation time of 20 min was selected in this work.

### 3.4. The Determination of Carbofuran

Under the optimal conditions, the immunosensor was incubated in carbofuran solutions of different concentrations. When the carbofuran was bound with the anti-carbofuran, the carbofuran was immobilized on the GCE. The carbofuran-antibody complex formed an additional layer to block the electron from solution diffusing to the surface of electrode, and thus the current response of the immunosensor decreased [26].

**Figure 4.**
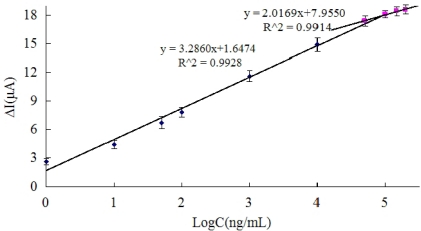
Calibration curve of the current response *vs.* the concentrations of carbofuran under optimal conditions.

Figure 4 displays the result of Ab/SiSG/GCE sensor incubated with the different concentrations of carbofuran for 20 min. The calibration plots relating the changes of current response (ΔI) and different concentrations of carbofuran are shown in Figure 4. The linear ranges were from 1 ng/mL to100 μg/mL and from 50 μg/mL to 200 μg/mL. The detection limit was 0.33 ng/mL, which was calculated at a signal to noise ratio of 3. The detection limit obtained is significantly lower than that reported (25 ng/mL) as the detection limit of ELISA determination of carbofuran [27], and it is comparable with that reported simple sensitive spot optical test for the rapid one-shot detection of carbofuran [28], and also easier to control than labeled immunochromatographic assay for carbofuran detection [29], indicating that the proposed Ab/SiSG/GCE immunosensor is reliable for the determination of carbofuran pesticides.

Figure 4 displays the result of Ab/SiSG/GCE sensor incubated with the different concentrations of carbofuran for 20 min. The calibration plots relating the changes of current response (ΔI) and different concentrations of carbofuran are shown in Figure 4. The linear ranges were from 1 ng/mL to100 μg/mL and from 50 μg/mL to 200 μg/mL. The detection limit was 0.33 ng/mL, which was calculated at a signal to noise ratio of 3. The detection limit obtained is significantly lower than that reported (25 ng/mL) as the detection limit of ELISA determination of carbofuran [27], and it is comparable with that reported simple sensitive spot optical test for the rapid one-shot detection of carbofuran [28], and also easier to control than labeled immunochromatographic assay for carbofuran detection [29], indicating that the proposed Ab/SiSG/GCE immunosensor is reliable for the determination of carbofuran pesticides.

### 3.5. Regeneration and Stability of the Immunosensor

**Figure 5.**
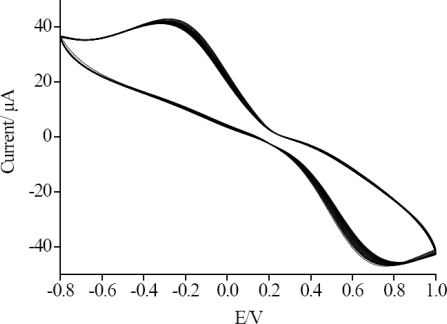
60 cycles of CV measurements of Ab/SiSG/GCE in 5 mmol/L [Fe(CN)_6_]^3−/4−^ solution.

After the proposed immunosensor was incubated in 10 μg/mL carbofuran for 20 min, it was dipped into a glycine-HCl buffer (pH 2.8) for about 5 min to remove carbofuran, and washed with water. Consecutive measurements were repeated for four times and the relative standard deviations (RSD) were 5.3% (n = 5). Under the optimal conditions, the immunosensor was measured by CV for 60 successive scan cycles, and a 2.7% deviation of the initial response was observed (Figure 5). The stability of the long-term storage to the Ab/SiSG/GCE sensor was evaluated by measuring the current response for 10 days. The prepared immunosensors were suspended in the PBS at 4 °C, and measured every day, during the 10 days storage it retained 93.2% of their initial response.

### 3.6. Specificity of the Immunosensor

**Figure 6.**
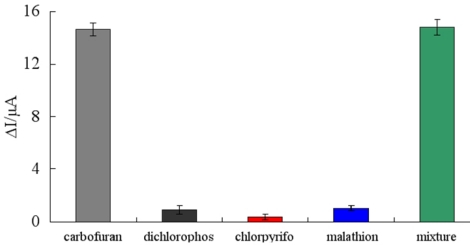
Specificity of immunosensor in the presence of dichlorophos (10 μg/mL), chlorpyrifos (10 μg/mL), malathion (10 μg/mL), carbofuran (10 μg/mL) and the mixture of carbofuran and the three kinds of interferences stated above.

In order to investigate the sensitivity of the immunosensor against the interferences arising from other non-target compounds that are expected to exist in real samples, we chose dichlorophos, chlorpyrifos, and malathion as interfering substances to evaluate the specificity of the immunosensor. Possible interferences were investigated by detecting the amperometric responses in 10 μg/mL carbofuran, 10 μg/mL dichlorophos, 10 μg/mL chlorpyrifos, 10 μg/mL malathion and the mixture of all four kinds of pesticides, respectively (Figure 6). According to the experiments, the ΔI respectively showed 14.64 μA, 0.9 μA, 0.34 μA, 1.04 μA and 14.82 μA, indicating that the specificity of immunosensor was satisfactory.

### 3.7. The Detection of the Real Samples

To further demonstrate the practicality of the proposed immunosensor, the contents of carbofuran in real samples of cabbage and lettuce were detected by Ab/SiSG/GCE. The recovery test was studied by adding different amounts of carbofuran to three cabbage and lettuce samples, respectively. The recoveries ranged from 89.14% to 106.8%. The performance of the immunosensor is significantly better than that detection of carbofuran by the AChE/PAMAM-Au/CNTs sensor [22], and is more stable than the performance of the enzyme-linked immunoassay [30]. Moreover, the performance of proposed immunosensor, was also compared with others for the detection of carbofuran in Table 1. The results indicated that the proposed method was highly accurate, precise and reproducible. It can be used for direct analysis of practical samples.

## 4. Conclusions

In this paper, a label-free immunosensor based on silica sol-gel/antibody composite film is developed for carbofuran detection. The proposed immunosensor is very simple to operate by direct immunoreaction detection without any label and competitive assay. Moreover, the silica sol-gel possesses a silicate network that provides a biocompatible microenvironment around the antibody, which can load larger amounts of antibody and thus display higher immunoactivity. The sensitivity of the sensor ensures its use, not only in the laboratory but also in the field for detection of carbofuran. Additionally, the immunosensor is low-lost and can be fabricated easily. It exhibits promising application prospects as a novel sensor.

## Figures and Tables

**Table 1.** The properties of this sensor compared with others reported.

**Sensors**	**Recovery (%)**	**References**
AChE/PAMAM-Au/CNTs	87.5	[22]
enzyme-linked immunoassay	74.55–123	[30]
Ab/SiSG/GCE	89.14–106.8	This work
